# Supplementary material for: UDP-Glucose: A Cereblon-Dependent Glucokinase Protein Degrader
Source: Int J Mol Sci. 2022 Aug 13;23(16):9094. doi: 10.3390/ijms23169094 (PMC9409010; doi:10.3390/ijms23169094)
Supplement: Supplementary file 1 [file ijms-23-09094-s001.zip › ijms-1816189-supplementary.pdf]

Supplemental information for

UDP-Glucose: A Cereblon-Dependent Glucokinase Protein Degrader.

This pdf file includes:

Abbreviations

Supplementary Figures S1 to S6

Abbreviations

BSP, bromosulfophthalein

cIAP1, cellular inhibitor of apoptosis protein 1

CRBN, cereblon

CRBN-F, FLAG-tagged cereblon

CRL4, cullin ring ubiquitin ligase 4

CUL4, cullin 4

DDB1, damage-specific DNA binding protein 1

2DG, 2-deoxy-ad-glucose

ER, endoplasmic reticulum

GCK, glucokinase

GCK-HA, HA-tagged glucokinase

GLUT, glucose transporter

GSIS, glucose-induced insulin secretion

IC_{50}, 50% inhibitory concentration

IMiDs, Immunomodulatory drugs

IP, immunoprecipitation

MDM2, murine double minute 2

MODY, maturity-onset diabetes of the young

PROTACs, proteolysis-targeting chimeras

TCA cycle, tricarboxylic acid cycle

UGGT, UDP-glucose:glycoprotein glucosyltransferase

UGP, UDP-glucose pyrophosphorylase

UPR, unfolded protein response

VHL, von Hippel-Lindau

WB, Western blot

WT, wild type

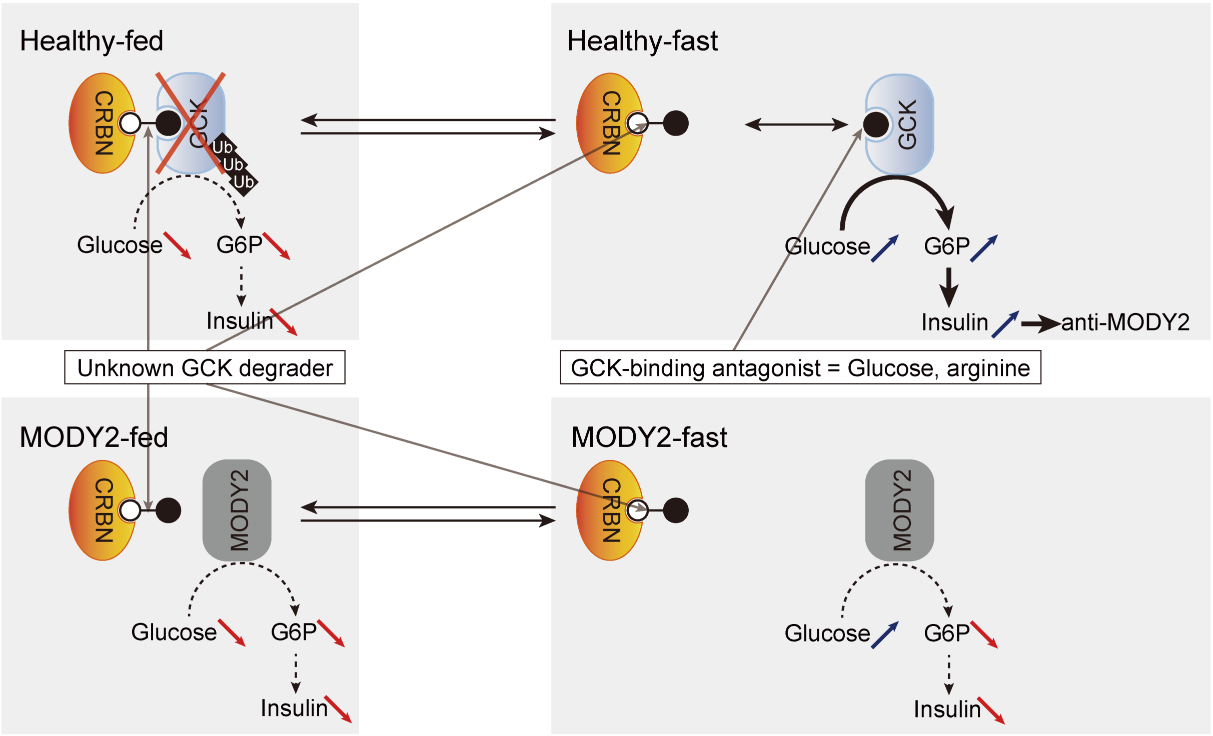

Supplementary Figure S1. Glucose inhibits glucokinase ubiquitination and degradation. (Supplementary data for Fig. 1).

(**S1a**) Summary of Fig 1. During fasting, the unknown glucokinase protein degrader X degrades glucokinase, and G6P production is reduced. After feeding, glucose and arginine bind to glucokinase and repress X-dependent glucokinase degradation, leading to induction of G6P production. In the case of glucokinase variants of MODY2, glucokinase variants less bound to glucose, and less G6P production.

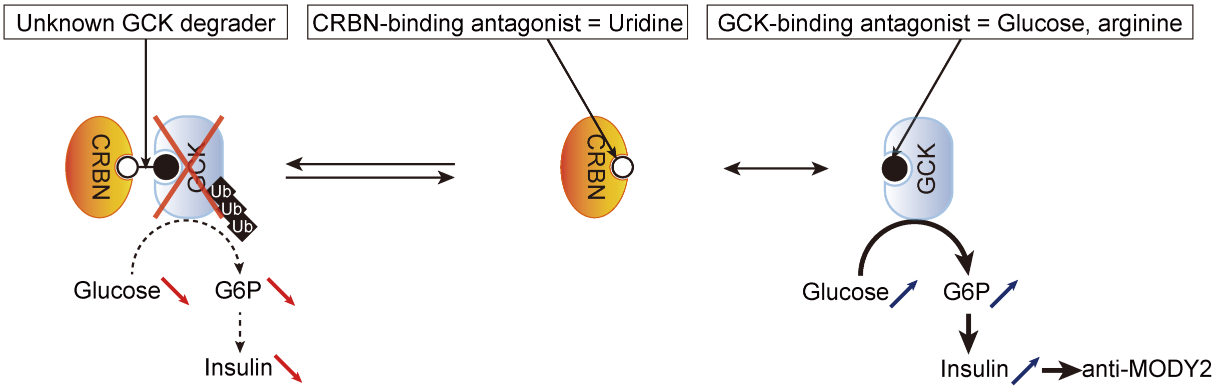

Supplementary Figure S2. Uridine binds to cereblon (CRBN). (Supplementary data for Figure 2).

Possible model of glucokinase cereblon-dependent ubiquitination and degradation by endogenous ligands.

Agonist; unknown

CRBN-binding antagonist; uridine

Glucokinase-binding antagonist; glucose, arginine

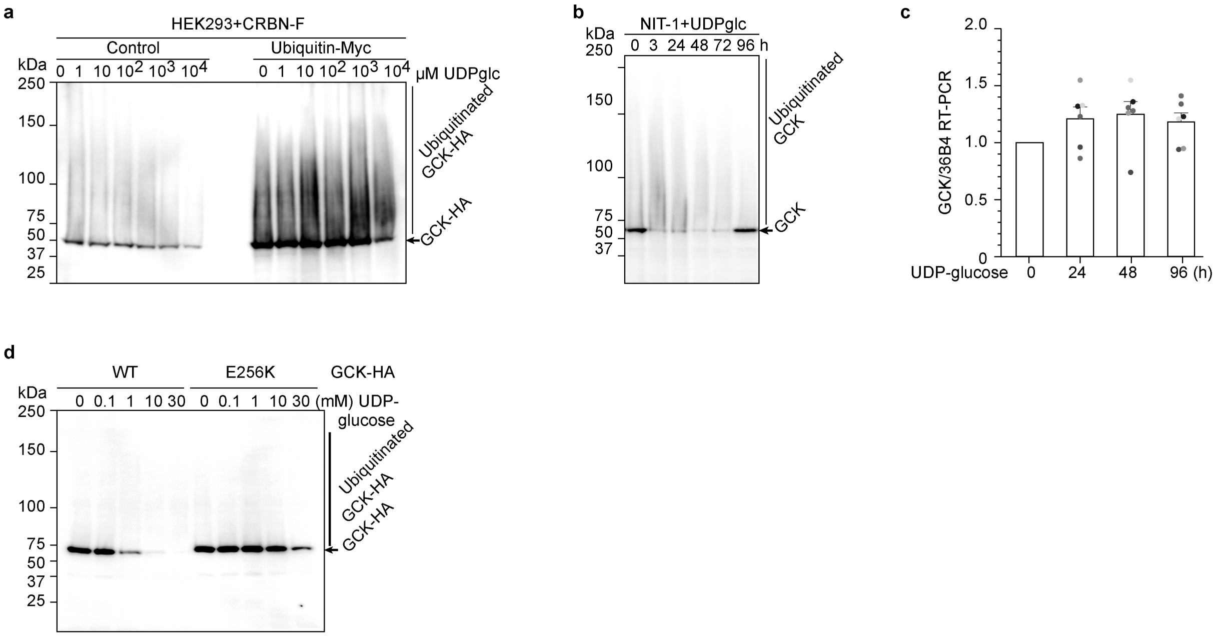

Supplementary Figure S3. UDP-glucose induces glucokinase ubiquitination and degradation. (Supplementary data for Fig. 3).

(**S3a**) UDP-glucose induces glucokinase ubiquitination in a dose-dependent manner. The HEK 293 cells were transfected with HA-tagged glucokinase (GCK-HA), FLAG-tagged cereblon (CRBN-F), and Myc-tagged ubiquitin (ubi-Myc) expression vectors for one day, and then UDP-glucose was added for 3 h. The cells were harvested and intracellular GCK-HA was detected by WB.

(**S3b**) UDP-glucose administration induced glucokinase degradation from 3 h to 72 h. NIT-1 cells were treated with UDP-glucose for the indicated times (0, 3, 24, 48, 72, and 96 h). Ubiquitinated and non-ubiquitinated glucokinase were detected with anti-glucokinase WB.

(**S3c**) UDP-glucose administration does not change glucokinase transcription. NIT-1 cells were treated with UDP-glucose for 0, 24, 48, and 96 h. Total RNA was extracted, and RT-PCR was performed.

(**S3d**) One mM UDP-glucose administration induced glucokinase^{WT} ubiquitination and degradation, although thirty mM UDP-glucose induced glucokinase^{E256K} mutated protein.

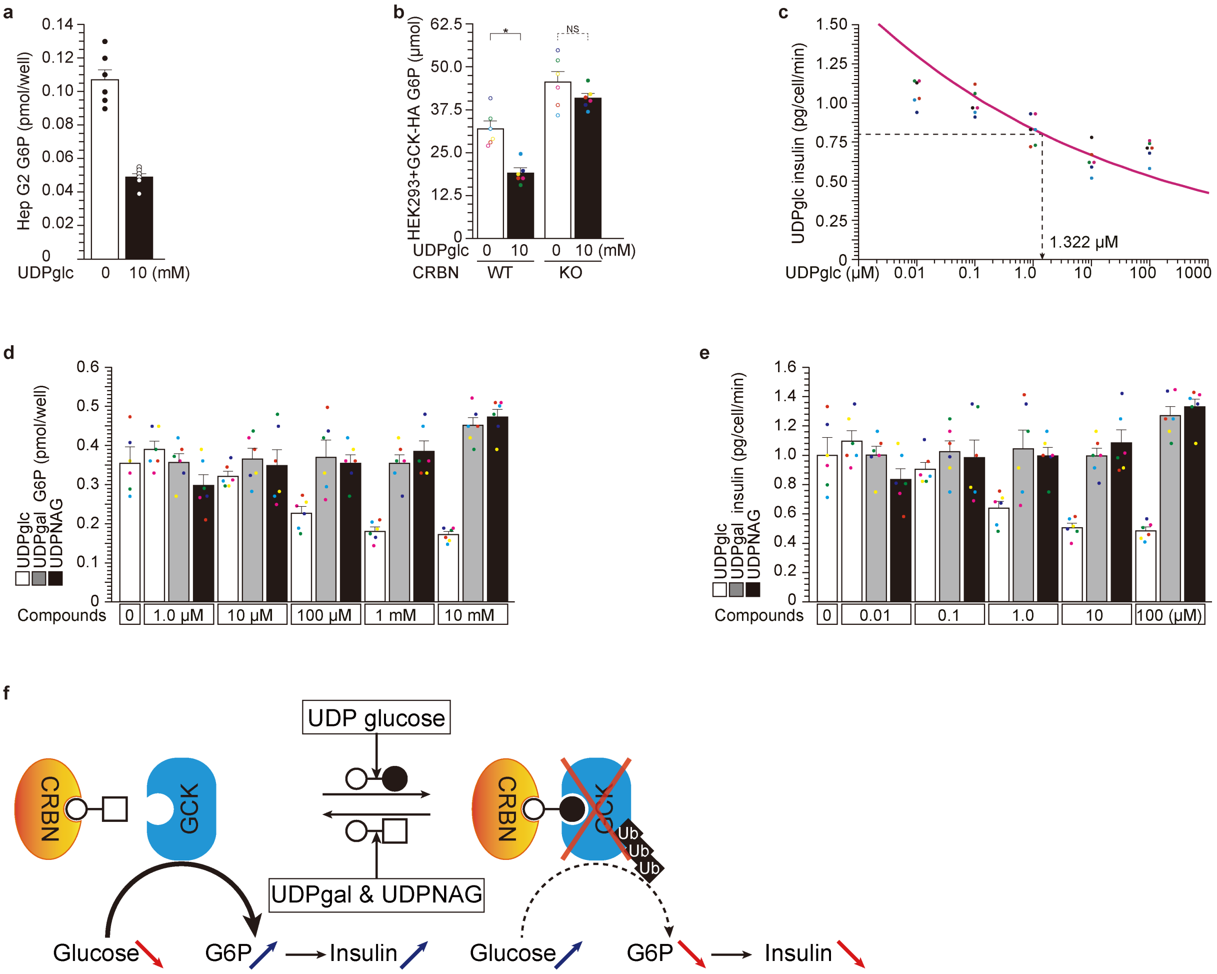

Supplementary Figure S4. UDP-glucose induces glucokinase degradation, and reduces insulin secretion from 3 h to 24 h. (Supplementary data for Fig. 4).

(**S4a**) UDP-glucose administration reduced G6P production in Hep G2 cells for 3 h, and then the cells were extracted. Intracellular G6P was analyzed using commercial kit.

(**S4b**) UDP-glucose did not reduce G6P production in CRBN KO cells for 3 h, and then cells were extracted. Intracellular G6P was analyzed using commercial kit. *; $p<0.05$, NS; $p>0.05$.

(**S4c**) UDP-glucose degrades proinsulin at IC_{50} = 1.322 μM

(**S4d** and **e**) UDP-monosaccharide regulates G6P production and insulin secretion.

(**S4f**) Possible model of UDP-monosaccharide regulating glucokinase ubiquitination.

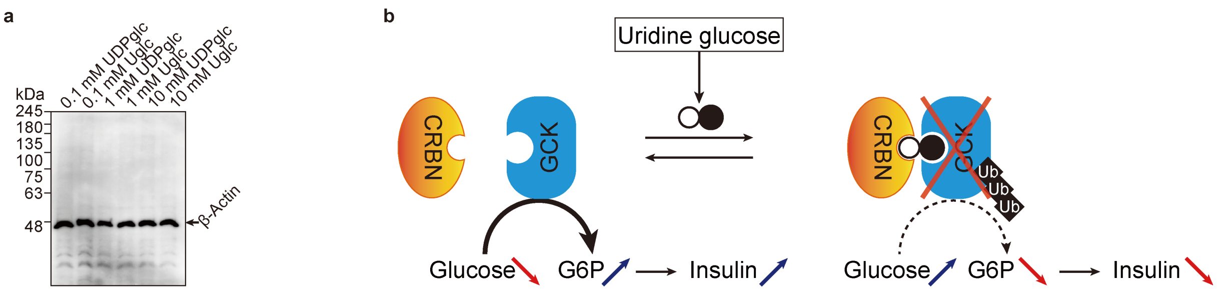

Supplementary Figure S5 Uridine-glucose degraded glucokinase, and reduced insulin secretion. (Supplementary data for Fig. 5).

(**S5a**) WB of control β-actin in Fig 5b.

(**S5b**) Possible model of uridine-glucose-degraded glucokinase protein.

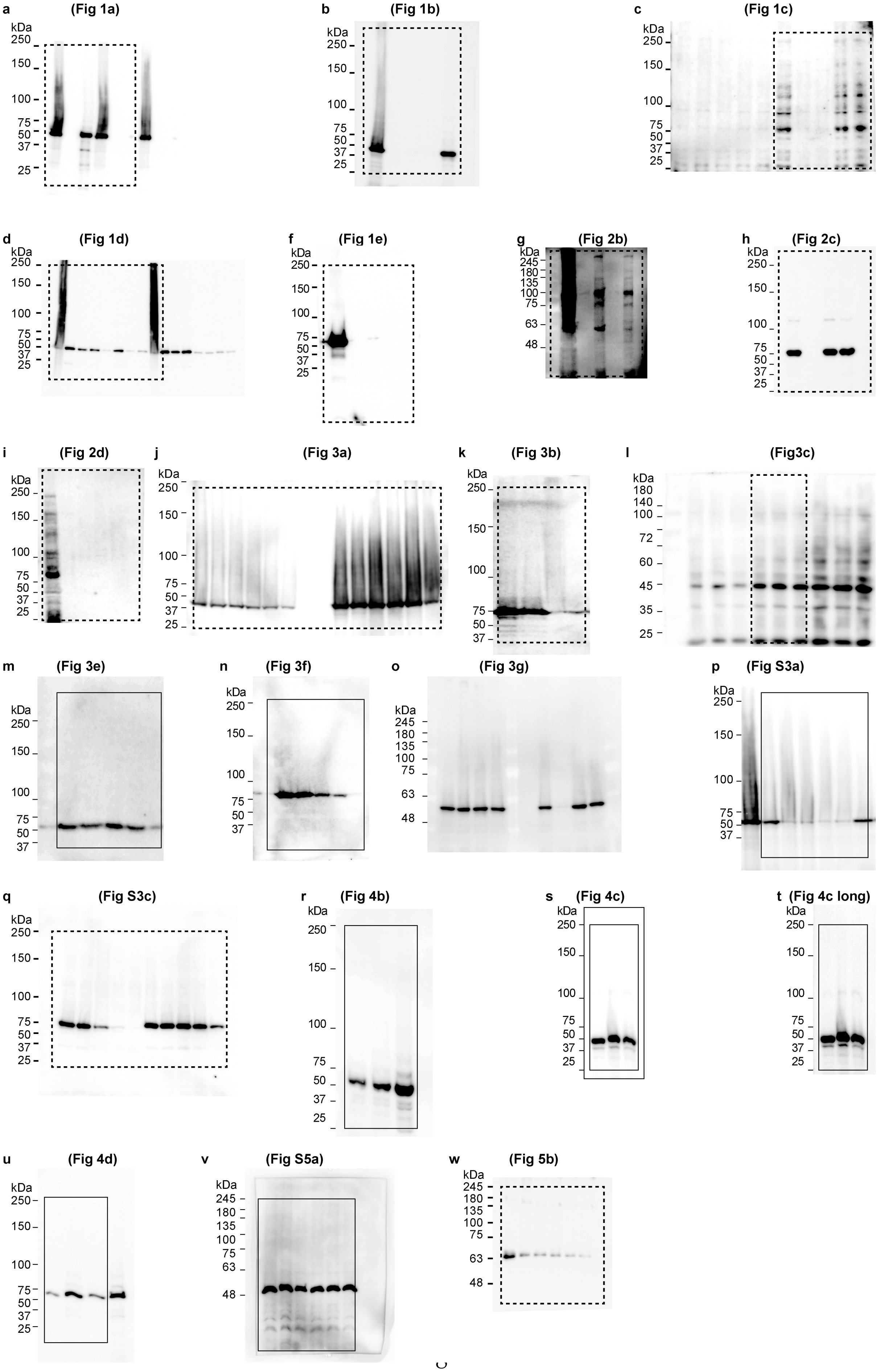
a (Fig 1a)
b (Fig 1b)
c (Fig 1c)
d (Fig 1d)
f (Fig 1e)
g (Fig 2b)
h (Fig 2c)
i (Fig 2d)
j (Fig 3a)
k (Fig 3b)
l (Fig3c)
m (Fig 3e)
n (Fig 3f)
o (Fig 3g)
p (Fig S3a)
q (Fig S3c)
r (Fig 4b)
s (Fig 4c)
t (Fig 4c long)
u (Fig 4d)
v (Fig S5a)
w (Fig 5b)
kDa

Figure S6 Source of Data
